# Supplementary material for: Evaluation of the hoof centre-of-pressure path in horses affected by chronic osteoarthritic pain
Source: PLoS One. 2023 Sep 15;18(9):e0291630. doi: 10.1371/journal.pone.0291630 (PMC10503732; doi:10.1371/journal.pone.0291630)
Supplement: Attachment — Submitted filename: Answer to Reviewer Definitiv document 25.08.2023.docx [file pone.0291630.s006.docx]

Reviewer #1: Thank you for attending to my comments on the previous version of the manuscript. The revised version is substantially improved. However, I have a few additional comments, see below.

Dear reviewer, thank you very much for considering our manuscript again and giving us further suggestions for improvement, we are very grateful for this. We will try to remove the last ambiguities in order to improve the manuscript even further.

Regarding calibration of the measurement equipment, my concern for lack of validation was referring to the pressure measurement equipment. The inertial measurement unit system used for assessment of movement asymmetries I don’t have any issues with. Sorry for being unclear.

Regarding calibration of the pressure measurement equipment, this is actually relevant even if you are only interested in the pressure distribution. The reason for this is that the output from the sensor isn’t linear with respect to the applied force, which means that the relative pressure distribution will be more accurate if calculated from calibrated values rather than raw voltages. This conference paper shows a graph nicely illustrating the nonlinearity of Tekscan sensors:
https://isbweb.org/images/conf/2005/abstracts/0263.pdf
Apart from calibration, Tekscan manual also recommends that you perform equilibration of the sensor. This is because the voltage output from each sensing element isn’t perfectly the same. In my experience, this is not a huge issue with a completely new sensor. However, with repeated use sensing elements that are subjected to high pressures become less sensitive to pressure over time. But I assume that the sensors you used were single use only?

Thank you for addressing this important issue. Indeed, right from the beginning, it induced a lot of thoughts from our side even if in the manuscript we did not present and discuss it properly. We correct the method section, add a sentence in the limitations and will explain here below our original thoughts and considerations.

When we started using the Tekscan in field situations, we got the suggestion to use the walk calibration method from researchers who previously used the sensors in horses. In their published papers, the calibration method was never really described in detail, it was just mentioned that the calibration was performed according to manufacturer recommendations. We soon realised that there is missing knowledge around this issue, that there are no generally accepted methods for field use and that the walk calibration is not adequate for accurate pressure measurements in case of gait asymmetries. While indeed we conditioned the sensors as suggested and calibrated them before data recordings using walk calibration on each individual horse, we then went back to uncalibrated force output to describe pressure distribution, as you know from our current manuscript. In the course of our literature search, we came across the Brimacombe paper (the full publication of the abstract you also mentioned), and once more, this convinced us that the calibration methods have a large role in the variability and errors of the pressure displayed by the Tekscan when compared to loads created from a materials testing machine. The authors suggest recording the force data as raw (uncalibrated) values and running a user-defined calibration algorithm offline, adequate for the range of pressures and experimental protocol used.

To make clear to the readers that we were not interested in absolute pressure data (but only in relative data, as provided by the Raw Sum display of the Tekscan), we stated that “we used an uncalibrated pressure measurement system”, which indeed is not correct, we merely used uncalibrated data for the purpose of the study. This sentence is now changed and we add the conditioning and walk calibration procedure in the description of the methods.

As you say, what we should have done to render the raw cells output perfectly homogeneous and to account for the small variation possibly existing among sensing elements would have been the equilibration procedure. For this purpose, a special device able to apply a very uniform pressure on the whole sensing surface is needed. We did not have it available at the time of data recording and we are convinced that the potential effect of this slight cells output variability on the COPp behaviour can be considered negligible. Indeed, COP derives from the sum of all forces, small variations among cells would be thus easily “compensated” and not further amplified.

Literature: Effect of Calibration Method on Tekscan Sensor Accuracy, Brimacombe et al 2009. DOI: 10.1115/1.3005165

Thank you for adding the movement asymmetry values to table 1. This clarifies my question of how the subjective and objective data were combined as both lameness degree and objective data are now available in this table.

Thank you for bringing this to our attention.

The description of the first Procrustes analysis in the methods section is still unclear to me. When you compare the shape of the single-stride COPp, I assume this is comparing strides for the same limb within the same measurement sequence? Please add this in the text to avoid any confusion. Further, you describe that you compare stride 1 to stride 2 and state that “to avoid measurement errors, the Procrustes analysis was always performed between COPp recorded during 2 strides”. If you want to study within-measurement variation, then you would want to compare all pairs of strides, basically the Cartesian product. Please add a motivation why you were only interested in differences between consecutive strides (stride 1 vs 2, 2 vs 3 and so on). Maybe you’re trying to say in the sentence cited above that you anticipated that measurement errors would be more influential if comparing strides further apart (due to for example small shifts of the sensor position relative to the hoof during the trial)?

Thank you for your comment. Regarding the Procrustes analysis of the individual strides, we will change it in the text. Regarding the comparison between strides, thank you for pointing out that it is not clear. We have indeed compared all the strides with each other and not just the consecutive ones. We realised that this was not written clearly enough, and we will change it accordingly in the manuscript.

The statistical analysis and the description in the statistics section are much improved from the previous version. Thank you for this. Please add whether repetition was included as a linear or as a categorical effect as both are possible/reasonable choices (depending on the data).

Repetition was included as a categorical effect because there were no different time periods to consider.

In the results section, when reporting the results from the linear mixed model please add the estimated marginal effects in addition to the P values.

We added this in the manuscript.
